# Ileocecal mucinous carcinoma misdiagnosed as incarcerated hernia: A case report

**DOI:** 10.1515/biol-2022-1002

**Published:** 2024-12-16

**Authors:** Xiang Ji, Meng Wang, Aihong Zhao, Jian Ding, Yunjie Zhang

**Affiliations:** First Clinical Medical College, Shandong University of Traditional Chinese Medicine, 16369 Jingshi Road, Jinan City, Shandong Province, China; Department of General Surgery, Affiliated Hospital of Shandong University of Traditional Chinese Medicine, 16369 Jingshi Road, Jinan, Shandong, China; Department of General Surgery, Linyi Traditional Chinese Medicine Hospital, 211 Jiefang Road, Lanshan District, Linyi City, Shandong Province, China

**Keywords:** mucinous carcinoma, diagnostic errors, case report, incarcerated hernia, ileocecal junction

## Abstract

Mucinous carcinoma is a rare clinical disease. Although well described in the literature, a mucinous carcinoma diagnosis is often difficult due to its atypical clinical presentation. We report a female patient with a right inguinal mass and ileocecal myxo carcinoma who was misdiagnosed as having a right incarcerated inguinal hernia invading the peritoneum incarcerated inguinal hernia and ileocecal myxo carcinoma. Intraoperative exploration of the mucous material occupying the patient’s right lower abdominal cavity and exclusion of right inguinal incarcerated hernia revealed the misdiagnosis. The first clinical manifestations of ileocecal mucinous carcinoma are often not readily apparent and may be misdiagnosed as an incarcerated inguinal hernia. When a color ultrasonography suggests an incarcerated inguinal hernia, an abdominal CT should be considered, and an enhanced CT should be performed as needed to observe the abdominal cavity. Ileocecal mucinous carcinoma should also be distinguished from other diseases with similar clinical manifestations. The patient had received conservative treatment for acute appendicitis, and it is recommended to conduct a B-ultrasound, CT, and other reviews after surgery. Clinicians should be aware of missed surgical opportunities following appendicitis caused by mucinous adenoma.

## 1 Introduction

Highly differentiated tubular adenocarcinoma and papillary adenocarcinoma are more common than mucinous adenocarcinoma in the ileocecal region [1]. Mucinous adenocarcinoma refers to a tumor with at least 50% extracellular mucus, preferentially located in the sigmoid colon of the rectum [2]. Mucinous colorectal malignant adenomas are found in 10–20% of colorectal cancer patients [3,4] and are more common in women and younger patients; mucinous colorectal malignant adenomas are also more common in the proximal colon and are typically diagnosed at an advanced stage [5,6,7]. We report a female patient who experienced a misdiagnosis of a right inguinal mass caused by ileocecal mucinous carcinoma invading the peritoneum as a right inguinal incarcerated hernia. During the operation, mucinous substances occupied the patient’s right lower abdominal cavity, and hard masses could be reached in the blind part of the right lower abdominal gyrus; these masses invaded the omentum, right posterior peritoneum, right lateral peritoneum, right lower abdominal wall, and pelvic cavity, and these were fixed and immobilized. Multiple mucoid substances were found, which were nonresectable, so tumor reduction was performed. The patient recovered after surgery.

## 2 Case presentation

### 2.1 Patient history

The patient is a 75-year-old female. Due to the location of the tumor in the “right groin area” and the persistence of the tumor beyond 10 days, the patient was hospitalized. The patient was found to have a small, painless right groin area retractable mass without obvious inducement 1 year ago, which was prominent when standing, walking, and when abdominal pressure increased. No relevant treatment was given at that time. Ten days before admission, the tumor became prominent and could not be retracted, accompanied by abdominal pain and distension. Levofloxacin was taken orally for 2 days (500 mg/day) to prevent infection, but abdominal pain and distension were not significantly relieved. When the patient was admitted to the emergency department of the hospital, the results of an abdominal routine scan suggested an incarcerated hernia. After admission, the tumor in the right groin area was investigated and could not be retracted, accompanied by abdominal pain and distension, fever of up to 38.5°C, difficult stool, no fart and excrement within 3 days, and nearly 2.5 kg weight loss over the 6 month period before admission.


**Informed consent:** Informed consent has been obtained from all individuals included in this study.
**Ethical approval:** The research related to human use has been complied with all the relevant national regulations, institutional policies, and in accordance with the tenets of the Helsinki Declaration, and has been approved by the authors’ institutional review board or equivalent committee.

### 2.2 Physical and laboratory exams

The physical examination results revealed a fever of 38.5°C, heart rate of 109 times/min, respiration of 21 times/min, and blood pressure of 105/58 mmHg. A bulging mass of about 20 × 15 cm in size was observed in the patient’s right groin area, with red skin color, a clear border, and tough quality. The right groin mass was tender and could not be retracted, the bowel sound was weakened, and no abnormalities were observed in the left groin area. Laboratory examination showed the concentrations of White blood cells at 13.18 × 10^9^/L, neutrophils absolute value at 12.17 × 10^9^/L, Red blood cells at 2.3 × 10^12^/L, hemoglobin at 74 g/L, and hypersensitive C-reactive protein at >200 mg/L.

### 2.3 Surgical intervention and prognosis

**Figure 1:**
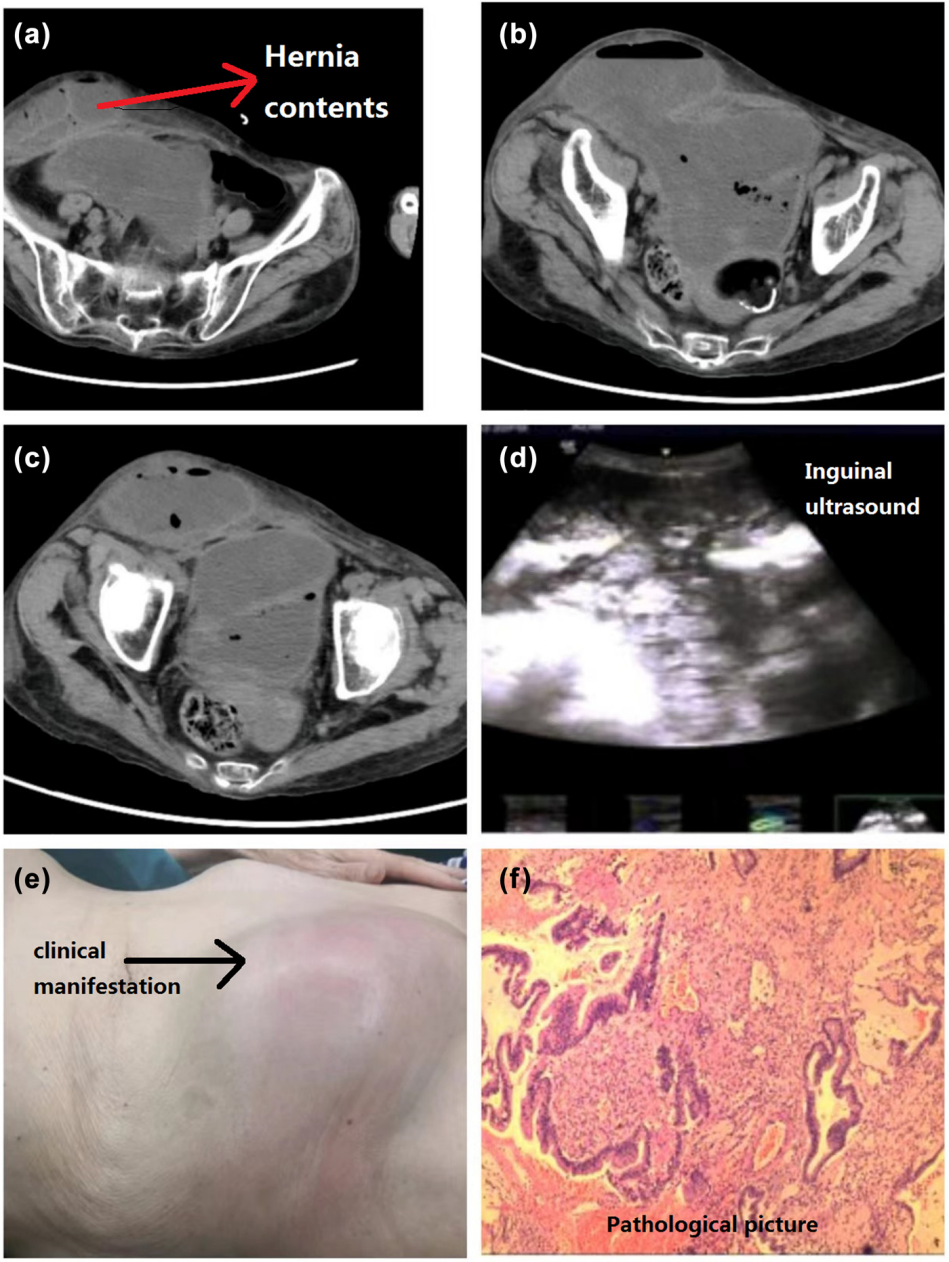
(a–c) Hernia contents; (d) inguinal ultrasound; (e) clinical manifestation; and (f) pathological picture.

A right inguinal incarcerated hernia restoration and exploratory laparotomy were prepared after the contraindication was excluded. During the operation, an oblique incision about 6 cm long was made in the right groin area, and the skin and subcutaneous tissue were cut. Edema and thin subcutaneous tissue showed subcutaneous purulent exudation; no hernia sac structure was seen, the structure of the inguinal canal was disturbed, and the abdominal wall of the lower right abdomen showed a huge pus cavity. After the incision, about 300 mL of grayish white pus was extracted. The pus cavity was about 15 × 12 cm in size and was cleaned up. A 2 × 3 cm abdominal wall defect at the base of the pus cavity was investigated and connected with the abdominal cavity down to the pelvic cavity. Mucinous tumor invasion of the right lower abdomen was considered. After the medical team explained the situation to the patient’s family, the patient underwent exploratory abdominal resection, reduction of mucinous tumor in the ileocecal junction, and incision debridement and drainage for infection of the right lower abdominal wall. About 100 mL of ascites was detected in the abdomen through the rectus abdominis exploration incision, and hard masses could be reached in the blind part of the right lower abdomen that invaded the omentum, right posterior peritoneum, right lateral peritoneum, right lower abdomen abdominal wall, and pelvic cavity; these masses were fixed and immobilized. Multiple mucoid substances were found and could not be resected. After mucous cleaning, tumor reduction surgery was performed. No intestinal content overflow was found, and the excision was sent to routine pathology. Pathological findings showed that the morphology was consistent with mucinous carcinoma secondary to low-grade mucinous tumor, and primary teratoma of the small intestine, appendix, and ovary were excluded. The patient was discharged successfully after surgery (Figure 1).

## 3 Discussion and conclusion

An incarcerated inguinal hernia is a common and frequently occurring disease in clinical surgery. Its main manifestations include abdominal pain, lack of appetite, nausea, vomiting, etc. In severe cases, the hernia block will suddenly harden and enlarge [8]. The annual prevalence rate of adult inguinal incarcerated hernia is estimated at 0.3–2.9%. Due to the narrow hernia ring and the failure of hernia contents to return to the abdominal cavity, blood flow disturbance will occur. If not handled in a timely manner or if handled improperly, it will progress to a strangulated hernia, which may cause intestinal obstruction, necrosis, rupture, peritonitis, and toxic shock and may even be life-threatening in severe cases [9]. An incarcerated inguinal hernia generally occurs in middle-aged and elderly people. Once the disease occurs in patients, if it is not treated in time, it may cause intestinal obstruction, and intestinal perforation symptoms may occur in severe cases, eventually evolving into a strangulated hernia, which seriously affects the safety and quality of life of patients [10]. An incarcerated hernia is characterized by sudden enlargement of the hernia mass along with significant pain, failure to retract the hernia mass by lying flat or pushing by hand, and significant tenderness. The symptoms of mechanical intestinal obstruction, such as abdominal distension and stopping of fart and defecation, occurred. 10 days before admission, the patient had a prominent tumor that could not be retracted, accompanied by abdominal pain, distension, fever reaching 38.5°C, constipation, no exhaust and defecation in 3 days, and other symptoms. The tumor was protruding in the right groin, about 20 × 15 cm in size, red in color, clear in boundary, tough in quality, and tender; the mass was unretractable and there was weakened bowel sound. The Leucocyte rate was 13.18 × 10^9^/L, CT and color ultrasound and other related auxiliary examinations are helpful for the diagnosis of inguinal incarcerated hernia. Therefore, an incarcerated hernia could not be ruled out.

Clinical manifestations are not limited to increased stool frequency, cases of severe and long-term constipation have been described for tumors of the ileocecal region. Histological types are divided into papillary adenocarcinoma, tubular adenocarcinoma, mucinous adenocarcinoma, etc. The most common are highly differentiated tubular adenocarcinoma and papillary adenocarcinoma. Mucinous adenocarcinoma of the ileocecal region is rare [1]. Mucinous adenocarcinoma has a high malignancy, high recurrence rate, and high metastasis rate, and it is difficult to make a definitive diagnosis based on the preoperative colonoscopy pathology [11]. Adenocarcinoma of the appendix is a rare malignant invasive adenoid tumor, accounting for 0.08% of all surgically removed appendicitis cases and 0.2–0.5% of all gastrointestinal tumors [12,13,14,15,16]. Intraoperative exploration of palpable hard masses in the blind part of the right lower abdominal gyrus, fixed and immovable, combined with a previous history of appendicitis and pathological reports suggest that the primary site of the disease in this patient is the appendix, and it is considered to be mucous cancer secondary to a low-grade mucous tumor of the appendix. Pseudomyxoma peritonei (PMP) was caused from the perforation rupture, resulting from penetration of the abdominal wall. Prolonged appendicitis symptoms may indicate the presence of a malignant adenoma of the appendix [16]. When penetrating the wall of the appendix, mucinous tumors may attach the appendix to a neighboring organ (e.g., the psoas major muscle) and less commonly arise through the skin of the fistula. It may be diagnosed as a muscle abscess. Rupture of mucocele can lead to PMP [17,18].

Combined with the clinical characteristics of incarcerated hernia, the patient had prominent and unretractable masses 10 days before admission, accompanied by abdominal pain and distension, and no exhaust and defecation for 3 days. The patient had previously retractable masses and had a reversible history. The patient described symptoms of unretractable masses 10 days ago, so incarceration and secondary infection were considered. A bulging mass was found in the patient’s right groin area, about 20 × 15 cm in size, with red skin color, clear border, and tough quality. The right groin mass was tender and could not be retracted, and the intestinal bowel sound was weakened. No abnormalities were found in the left groin area. Combined with B ultrasonography and CT-assisted examination, the results were consistent with an incarcerated hernia. Combined with the relevant auxiliary examination and the patient’s symptoms, the patient was presumed to have the characteristics of an incarcerated hernia. Thus, our preliminary diagnosis was an incarcerated inguinal hernia. Combined with the patient’s history, characteristics, symptoms, and signs, the patient was considered to have a right inguinal incarcerated hernia. Due to the atypical signs and symptoms of ileocecal carcinoma, the patient did not complain of pus and bloody stools or increased stool frequency, so they were misdiagnosed with a right inguinal hernia.

Nagata et al. [19] reported a case of a large appendiceal mucocele characterized by abdominal mass. Handler et al. [20] reported a unique case of malignant mucinous adenoma of the appendix in a 57-year-old man, who initially presented with a mass in the right thigh followed by a tumor extending from the right lower abdomen to the femoral duct. There are similarities between this case and the one we have reported. Spiridakis et al. [21] reported a rare case of anal fistula and recurrent perianal abscess in a 79-year-old Greek Caucasian male patient, which was subsequently found to have developed synchronous recto-sigmoid and perianal mucous adenocarcinoma at biopsy. Histological examination revealed two sites of mucinous adenocarcinoma, representing two tumors. The patient underwent a combined laparoscopic perineal and extramural levator abdominectomy in which both lesions were removed. There was no recurrence after 4 years of follow-up. El Alaoui et al. [22] reported a case of mucinous adenocarcinoma of the scrotum secondary to the fistula of the scrotum. Stewart et al. [23] reported on a patient with metastatic malignant adenoma of the pancreas, which presented as cystic masses in the supraclavicular and axillary areas. Wang et al. [24] reported a case of mucinous adenocarcinoma of the ileocecal region with hip abscess as the first manifestation.

Mucocele ileocecal carcinoma is a rare clinical disease. In clinical cases, routine clinical manifestations include changes in bowel frequency, duration of abdominal pain, nausea, and vomiting. Special clinical manifestations include surface mass, incarcerated hernia, anal fistula, and hip abscess. Ultrasound, CT, and other examinations should be taken into account in the diagnosis, and a biopsy should be performed if necessary to confirm the diagnosis. Pay attention to patients’ previous abdominal history and be aware of missed surgery opportunities due to myxadenoma-related diseases such as appendicitis.
